# Epidemiology, Risk Factors, and Outcomes of Out-of-Hospital Cardiac Arrest Caused by Stroke: A Population-Based Study

**DOI:** 10.1097/MD.0000000000003107

**Published:** 2016-04-08

**Authors:** Tatsuma Fukuda, Naoko Ohashi-Fukuda, Yutaka Kondo, Toshiki Sera, Kent Doi, Naoki Yahagi

**Affiliations:** From the Department of Emergency and Critical Care Medicine, Graduate School of Medicine, The University of Tokyo, Bunkyo-ku, Tokyo, Japan (TF, NO-F, KD, NY); Division of Acute Care Surgery, Trauma, and Surgical Critical Care, Department of Surgery, Beth Israel Deaconess Medical Center, Harvard Medical School, Boston, MA, USA (YK); and Department of Acute Critical Care and Disaster Medicine, Tokyo Medical and Dental University, Bunkyo-ku, Tokyo, Japan (TS).

## Abstract

Limited information is available regarding stroke-related out-of-hospital cardiac arrest (OHCA). We aimed to assess the clinical characteristics of stroke-related OHCA and to identify the factors associated with patient outcomes.

We conducted a nationwide population-based study of adult OHCA patients in Japan from January 1, 2006 to December 31, 2009. We examined the epidemiology, risk factors, and outcomes of stroke-related OHCA compared with cardiogenic OHCA. The primary outcome was neurologically favorable survival.

Of the 243,140 eligible patients, 18,682 (7.7%) were diagnosed with stroke-related OHCA. Compared to OHCA with a presumed cardiac etiology, stroke-related OHCA patients had a greater chance of prehospital return of spontaneous circulation (ROSC) (9.9% vs 5.9%, *P* < 0.0001) but a reduced chance of 1-month survival (3.6% vs 4.9%, *P* < 0.0001) or favorable neurological outcomes (1.2% vs 2.6%, *P* < 0.0001). After adjusting for a variety of confounding factors, the prehospital ROSC rates were higher (adjusted OR 2.47, 95% confidence interval [CI] 2.34–2.62), but the neurologically favorable survival rates were lower (adjusted OR 0.66, 95%CI 0.57–0.76), among the stroke-related OHCA patients. In stroke-related OHCA cases, having a younger age, witness, and shockable 1st documented rhythm were associated with improved outcomes. Men had more favorable neurological outcomes. Seasonal or circadian factors had no critical impact on favorable neurological outcomes. Prehospital advanced life support techniques (i.e., epinephrine administration or advanced airway management) were not associated with favorable neurological outcomes.

Although stroke-related OHCA had lower 1-month survival rates and poorer neurological outcomes than cardiogenic OHCA, the rates were not considered to be medically futile. Characteristically, sex differences might impact neurologically favorable survival.

## Abstract 2

Supplemental Digital Content is available in the text

## INTRODUCTION

Stroke has remained one of the leading causes of death worldwide over the past decade, along with ischemic heart disease.^1^ Stroke and ischemic heart disease can lead to sudden death, as well as death in the long-term.^2–4^ The causes of out-of-hospital cardiac arrest (OHCA) are mainly cardiac (particularly, ischemic heart disease); therefore, most OHCA studies have focused on ischemic heart disease, and they have clarified several important facts so far.^5–9^ However, little is known regarding the characteristics of OHCA caused by stroke.^2,10^ The optimal therapeutic strategy for OHCA may vary greatly by etiology.^10–14^ Therefore, detailed knowledge is required of OHCA caused by stroke. The previous largest study (86 patients) was conducted to identify the unique features of OHCA of neurological causes to determine the optimal care provisions.^10^ The study concluded that resuscitative efforts might be futile because of the approximately 100% mortality rate and that supportive treatments might be reoriented toward organ donation. However, we believe that larger samples are required before reaching such an important conclusion.

This nationwide population-based study aimed to examine the epidemiology, risk factors, and outcomes of OHCA caused by stroke based on large patient samples in Japan. In addition, to better understand the nature of OHCA caused by stroke, this study also aimed to compare the characteristics of stroke-related OHCA with those of cardiogenic OHCA, which have been well-researched.

## METHODS

### Study Design and Participants

The All-Japan Utstein Registry is an ongoing, nationwide, population-based registry of all OHCA patients; it is maintained by the Fire and Disaster Management Agency (FDMA) through standardized Utstein-style data collection.^15,16^ The registry design has previously been described in detail.^14,17,18^ Briefly, all patients with confirmed OHCA (defined as the absence of a palpable central pulse, apnea, and unresponsiveness) of all causes and for whom resuscitation was attempted are identified and followed. In Japan, most OHCA patients are treated and transported to an emergency hospital because emergency medical services (EMS) personnel are not allowed to terminate resuscitation using their own judgment out-of-hospital, except in specific situations (e.g., decapitation, rigor mortis, livor mortis, or decomposition). Furthermore, advance directives, living wills, or do-not-resuscitate orders are not generally accepted in Japan. Data are collected from 3 sources that together define the continuum of emergency cardiac care: 119 dispatch centers, EMS agencies, and receiving hospitals. Data completeness and accuracy are ensured through rigorous certification of hospital staff and use of standardized software, with internal data checks.

The study population consists of 444,480 patients who were enrolled in the All-Japan Utstein Registry from January 1, 2006 to December 31, 2009. Data from adult patients aged 18 years or older with OHCA with stroke or cardiac causes were analyzed. Only those patients without an extremely long prehospital time were eligible (i.e., the time from call to scene arrival and contact with patient was ≤120 minutes, the time from scene arrival and contact with patient to hospital arrival was ≤120 minutes, and the time from call to hospital arrival was ≤240 minutes) because patients who require an extremely long prehospital time may have distinct prehospital circumstances and outcomes. Patients with missing or unknown data such as age, etiology of cardiac arrest, date and time, or prehospital information (e.g., bystander status or prehospital care) were also excluded from the analysis. Missing or unknown data accounted for less than 10% of the total number of patients.

This study was conducted in accordance with the amended Declaration of Helsinki. The FDMA and the Institutional Review Board of The University of Tokyo approved the study, with an informed consent waiver because of the anonymous nature of the data (No. 10096).

### Procedures

Data were prospectively collected using an Utstein-style template that included information on age, sex, etiology of cardiac arrest, bystander witness status, bystander cardiopulmonary resuscitation (CPR) status, use of a public-access automated external defibrillator, 1st documented rhythm, presence of an emergency lifesaving technician or physician in the ambulance, administration of epinephrine, implementation of advanced airway management, and return of spontaneous circulation (ROSC) before the hospital arrival (prehospital ROSC). The date and time of onset, a series of EMS times (such as the call receipt, vehicle arrival at the scene, contact with patients, initiation of CPR, and hospital arrival), and the ROSC time were also recorded.

The etiology of OHCA was categorized as cardiac or noncardiac. Noncardiac causes were subdivided into respiratory disease, stroke, malignant tumor, external causes (e.g., trauma, drowning, burn, asphyxia, or intoxication), or other noncardiac causes. The etiology of OHCA was presumed to be cardiac unless evidence suggested a noncardiac cause.^16^ Therefore, the cardiac etiology included the confirmed and presumed cases. The OHCA etiology for survivors was probed during the hospital stays. However, the OHCA etiology for decedents was determined by the attending physicians in the emergency department, in collaboration with the EMS personnel or coroners, based on certain information, such as witness information, clinical course, past history, physical findings, examination findings, imaging, or autopsy. As autopsy rates are not high in Japan, the majority of OHCA cases were regarded as having presumed cardiac etiology.

The patients were followed up at 1 month by the EMS personnel in charge of each OHCA patient to collect data on 1-month survival and neurological status 1 month after the event, as well as OHCA etiology. The EMS personnel queried the medical control director at the hospital and received a written response. If the patient was not at the hospital, the EMS personnel conducted a follow-up investigation. The neurological status of each patient was determined by the attending physician who cared for the patient using Glasgow–Pittsburgh cerebral performance category (CPC) scores. A CPC score of 1 or 2 (good performance or moderate disability, respectively) was defined as a favorable neurological outcome, and a CPC of 3, 4, or 5 (severe disability, vegetative state, or death, respectively) was regarded as an unfavorable neurological outcome.^16,19^ Data forms were completed by the EMS personnel, and the data were integrated into the Utstein registry system on the FDMA database server. FDMA logically checked the data using the computer system and returned the data forms to the respective fire stations for reconfirmation, unless the data were complete.

### Statistical Analysis

We compared OHCA caused by stroke with OHCA caused by cardiac etiology to develop an understanding in contradistinction to well-researched OHCA. As many past OHCA studies have investigated presumed cardiac etiology cases, we compared stroke cases to overall presumed cardiac etiology cases. However, overall presumed cardiac etiology cases included a small proportion of confirmed cardiac etiology cases in Japan. Therefore, we also separately compared the stroke cases to the confirmed cardiac etiology cases.

The baseline characteristics of the study cohort were described with the use of proportions for categorical variables and the means with standard deviations for continuous variables. In the comparison, continuous variables were assessed through the analysis of variance, and categorical variables were assessed using the χ^2^ test.

To assess the factors associated with favorable outcomes, multivariable logistic regression analysis was used, and odds ratios (ORs) and their 95% confidence intervals (CIs) were calculated after adjusting for potential confounding factors that were biologically essential and assumed to be associated with the outcomes. These variables included age (18–64, 65–84, or ≥85 years), sex (male or female), witness (present or absent), bystander CPR (present or absent), public access automated external defibrillator use (present or absent), 1st documented rhythm (shockable or nonshockable), etiology of OHCA (stroke or cardiac), epinephrine administration (present or absent), advanced airway management (present or absent), physician in ambulance (present or absent), time from call to contact with the patient, time from contact with the patient to hospital arrival, season of onset (winter, spring, summer, or autumn), and time of onset (0:00–5:59, 6:00–11:59, 12:00–17:59, or 18:00–23:59). Multivariable logistic regression analysis was performed in 3 cohorts. One cohort consisted of only stroke cases, another consisted of stroke cases and overall presumed cardiac etiology cases, and the other consisted of stroke cases and only confirmed cardiac etiology cases.

All statistical analyses were conducted using JMP Pro 11.0.0 software (SAS institute Inc., Cary, NC). All tests were 2-sided, with a significance level of 0.05.

## RESULTS

**FIGURE 1:**
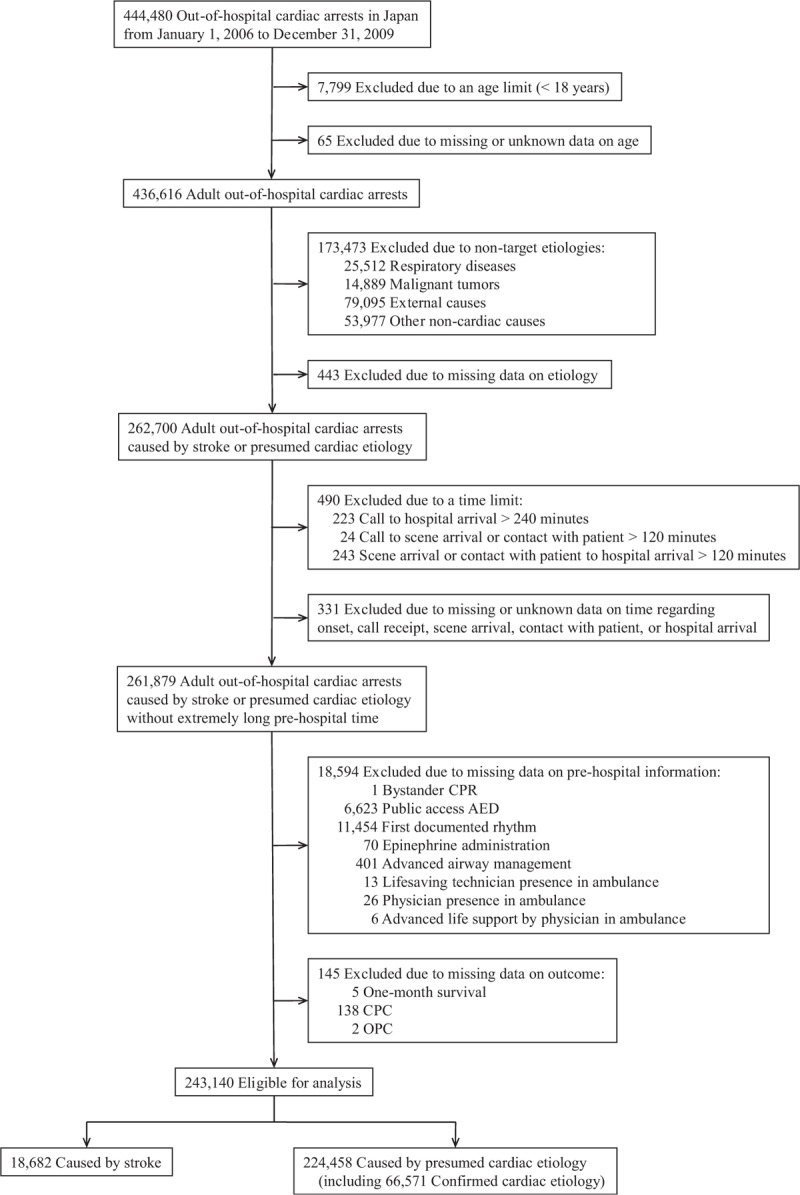
Study population. AED = automated external defibrillator, CPC = Glasgow–Pittsburgh cerebral performance category, CPR = cardiopulmonary resuscitation, OPC = Glasgow–Pittsburgh overall performance category.

Data for a total of 444,480 OHCA patients were submitted to the All-Japan Utstein Registry during the study period. Of these patients, 7864 were excluded based on age criteria (≥18 years), and 173,916 were excluded based on the etiology criteria (stroke or presumed cardiac etiology). Of the remaining 262,700 adult patients with OHCA caused by stroke or cardiac causes, 490 were also excluded because of extremely long prehospital time, which might imply simple input errors or distinct prehospital circumstances. In addition, the following patients were excluded because of missing or unknown data: 331 without date and time data (0.1%), 18,594 without data on prehospital information (7.1%), and 145 without outcomes data (0.1%). Finally, 243,140 patients were eligible for our analyses (Figure 1). Among these cases, 18,682 were caused by stroke, and 224,458 were caused by presumed cardiac etiology; 66,571 confirmed cardiac etiology cases accounted for approximately 30% of the overall presumed cardiac etiology cases (Figure 1).

Table 1 lists the baseline characteristics of each cohort. The mean age of the stroke patients was 71.6 years (standard deviation, 16.2 years), and the proportion of male patients was 50.9%. Compared to the cardiac etiology patients (both overall presumed and confirmed cases), the stroke patients were younger, and the proportions of male patients and those with shockable 1st documented rhythm were lower (*P* < 0.0001).

**TABLE 1:**
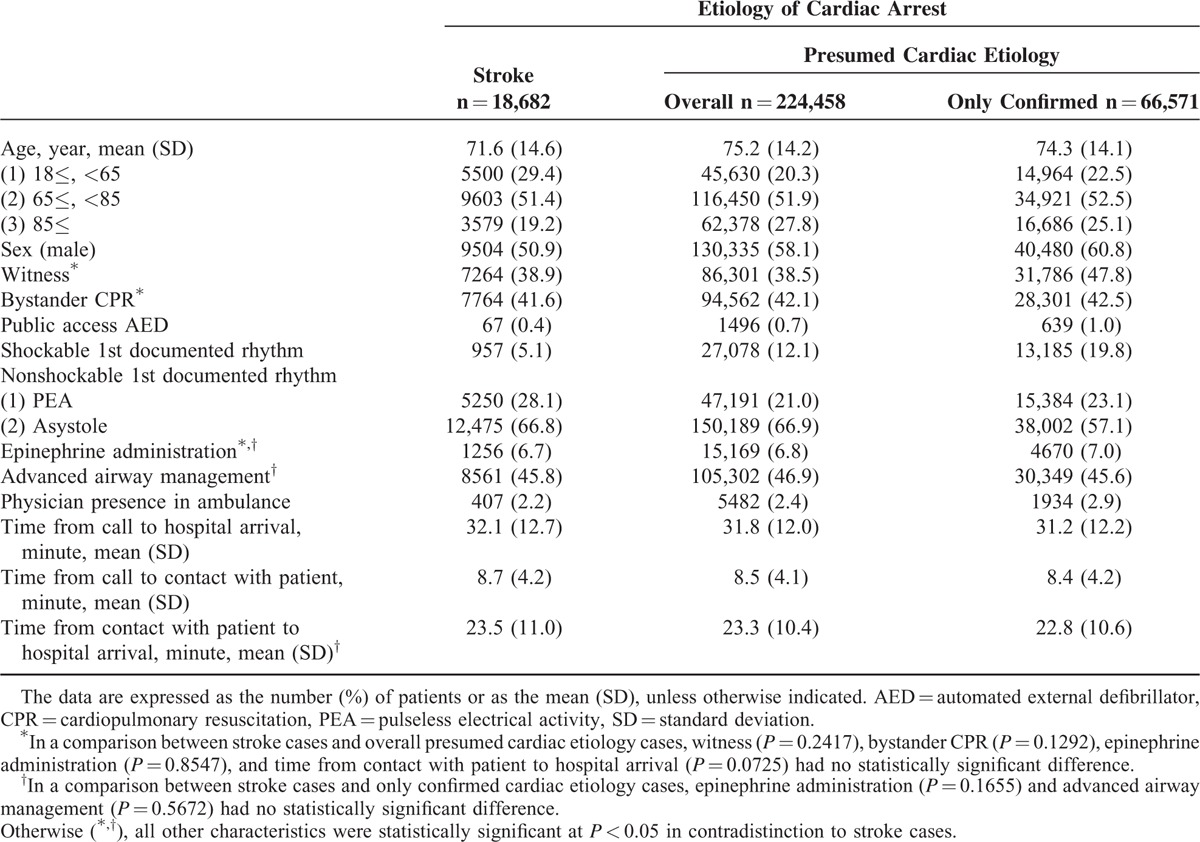
Baseline Characteristics

Table 2 shows the outcomes of each cohort. Although the proportion of prehospital ROSC among the stroke cases tended to be higher than that in cardiac etiology cases, the proportions of 1-month survival and favorable neurological outcomes were significantly lower. Compared with the cardiac etiology cases, in which more than half of 1-month survivors had favorable neurological outcomes, fewer than one-third of 1-month survivors had favorable neurological outcomes in the stroke cases.

**TABLE 2:**
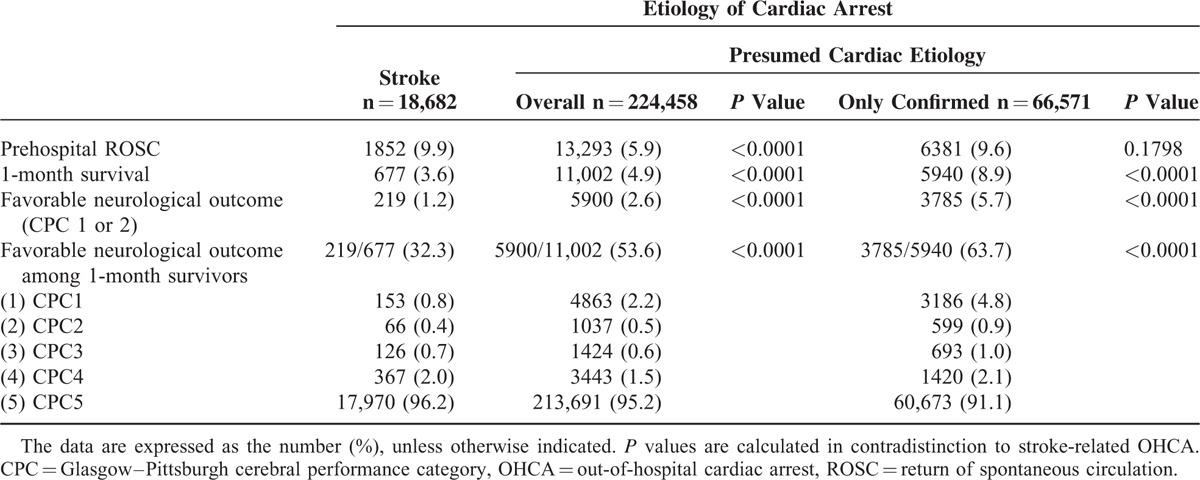
Outcomes

**FIGURE 2:**
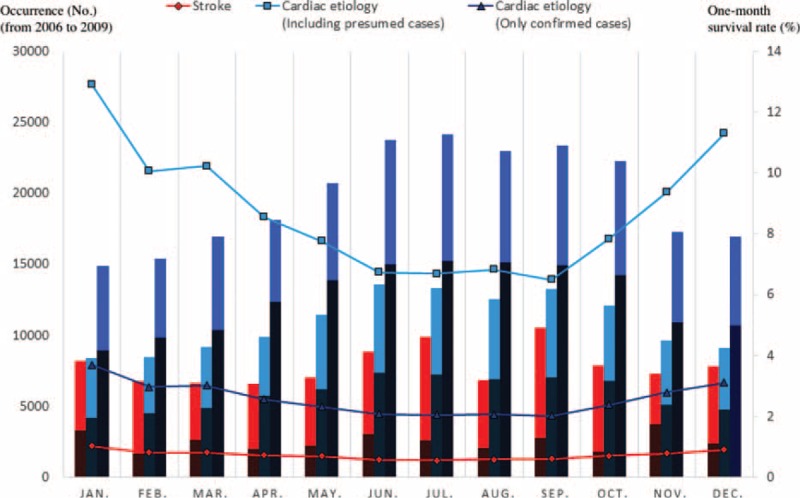
Occurrence, 1-month survival rate, and neurologically favorable survival rates of out-of-hospital cardiac arrest (OHCA) patients for each etiology by month of onset (from January 2006 to December 2009). The line graphs show the number of OHCA cases by month for 4 years. The bar graphs show the 1-month survival rate. The dark areas of the bar graph show favorable neurological outcomes, while the light areas show poor neurological outcomes.

**FIGURE 3:**
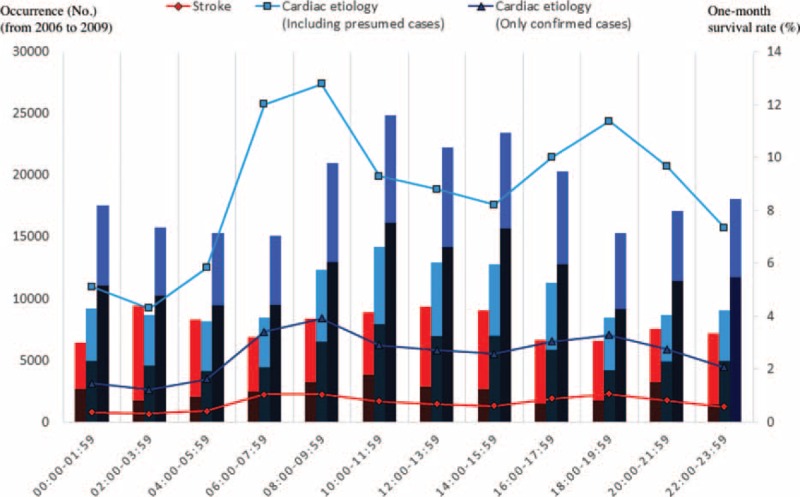
Occurrence, 1-month survival rate, and neurologically favorable survival rates of out-of-hospital cardiac arrest (OHCA) patients for each etiology by time of onset (from January 2006 to December 2009). The line graphs show the number of OHCA cases by time of onset for 4 years. The bar graphs show the 1-month survival rate. The dark areas of the bar graph show favorable neurological outcomes, while the light areas show poor neurological outcomes.

**FIGURE 4:**
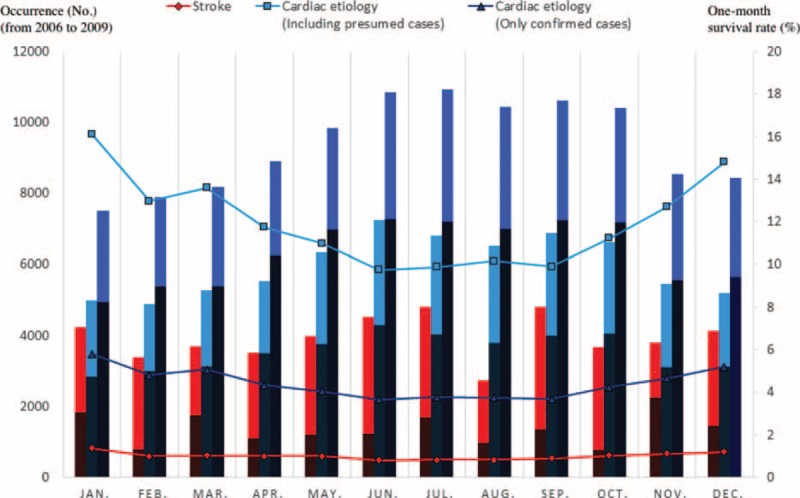
Occurrence, 1-month survival rate, and neurologically favorable survival rates of witnessed out-of-hospital cardiac arrest (OHCA) patients for each etiology by month of onset (from January 2006 to December 2009). The line graphs show the number of OHCA cases by month for 4 years. The bar graphs show the 1-month survival rate. The dark areas of the bar graph show favorable neurological outcomes, while the light areas show poor neurological outcomes.

**FIGURE 5:**
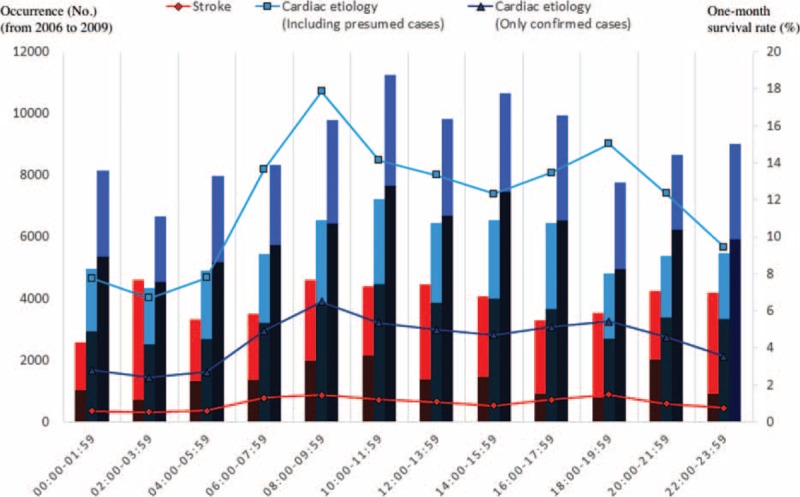
Occurrence, 1-month survival rate, and neurologically favorable survival rates of witnessed out-of-hospital cardiac arrest (OHCA) patients for each etiology by time of onset (from January 2006 to December 2009). The line graphs show the number of OHCA cases by time of onset for 4 years. The bar graphs show the 1-month survival rate. The dark areas of the bar graph show favorable neurological outcomes, while the light areas show poor neurological outcomes.

Figures 2 and 3 (and Appendices 1 and 2) show the occurrence, 1-month survival rate, and neurologically favorable survival rate of OHCA (from January 2006 to December 2009), according to each etiology by month or time of onset. Calls for ambulances occurred more frequently in the winter (January and December) and in the early morning (6:00–9:59) and late afternoon (16:00–19:59) in both the stroke and cardiac etiology cases. In the cardiac etiology cases (particularly confirmed cardiac etiology cases), the outcomes were more favorable when the ambulance call occurred in the summer or autumn (June–October) or during the day (10:00–15:59), seemingly in inverse proportion to the number of cases. For stroke cases, fixed tendencies were not observed between the outcomes and the month or time of the ambulance call. The time of the ambulance call does not necessarily represent the time at which the OHCA occurred, therefore, to directly examine the association between the onset time and outcome, an analysis that was limited to only the witnessed OHCA cases was also performed. The results are shown in Figures 4 and 5 (and Appendices 3 and 4). Even when limited to OHCA cases witnessed by bystanders, the numbers of cases and outcomes followed similar tendencies that were observed in the overall cohort.

Table 3 shows factors contributing to favorable outcomes in stroke-related OHCA. In stroke-related OHCA, having a younger age, a witness, shockable 1st documented rhythm, and a shorter time from call to contact with patient were associated with improving outcomes. Although men had more favorable neurological outcomes than women (adjusted OR 1.56, 95%CI 1.16–2.13), the prehospital ROSC rate was worse among men (adjusted OR 0.78, 95%CI 0.70–0.86). Although prehospital epinephrine administration and physician presence in the ambulance were associated with improved prehospital ROSC, they were not associated with 1-month survival or favorable neurological outcome. In addition, prehospital advanced airway management was associated with poor outcomes. Seasonal or circadian factors had no critical impact on 1-month survival or favorable neurological outcome in stroke-related OHCA. The factors that contributed to favorable outcomes in OHCA, including the stroke cases and overall presumed cardiac etiology cases, are shown in Table 4, and Table 5 includes the stroke cases and only the confirmed cardiac etiology cases. The prehospital ROSC rate was significantly higher in the stroke cases than in the cardiac etiology cases, although the 1-month survival rate and neurological outcomes were poorer among the stroke cases.

**TABLE 3:**
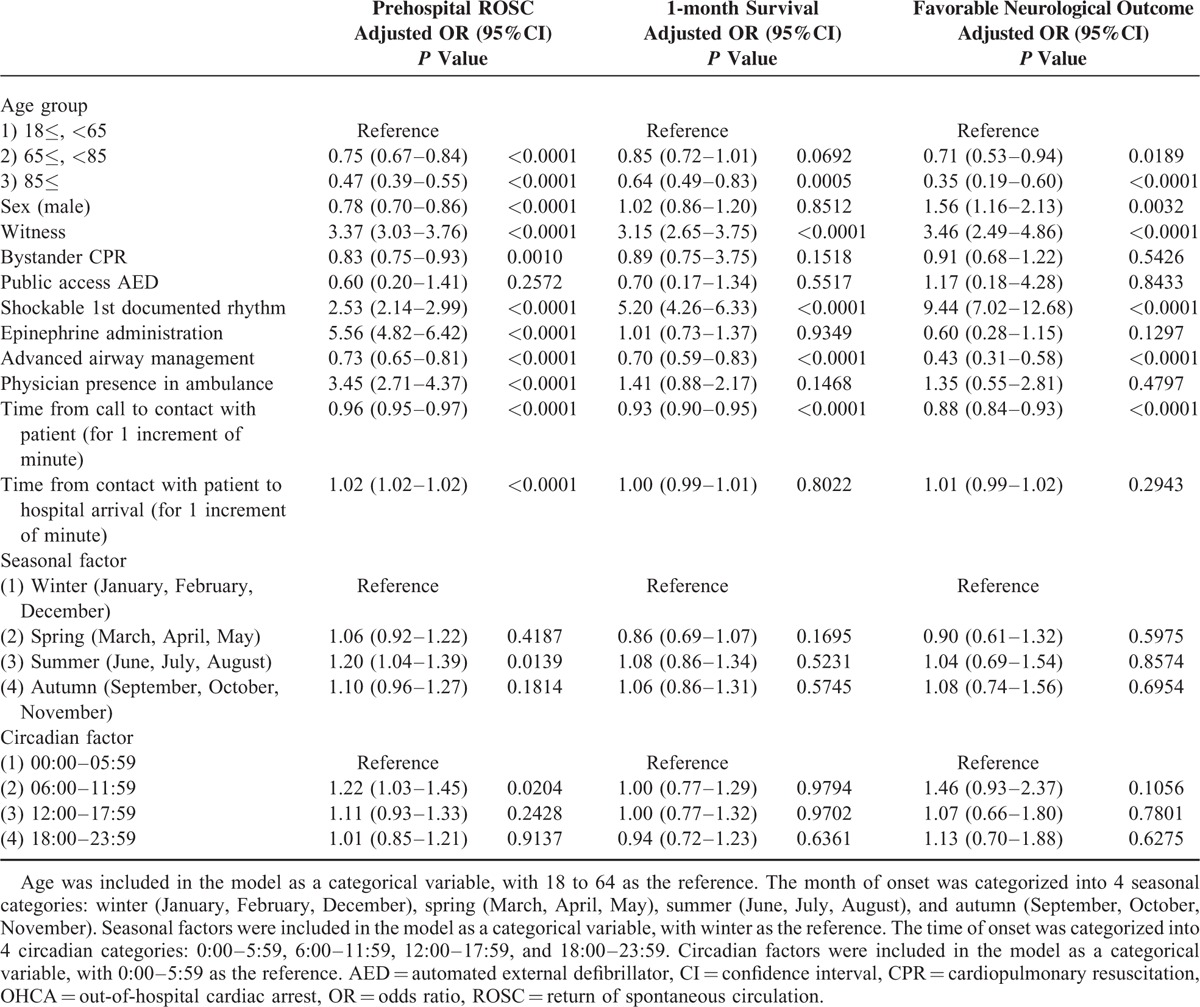
Factors Contributing to Favorable Outcomes in Stroke-Related OHCA Patients

**TABLE 4:**
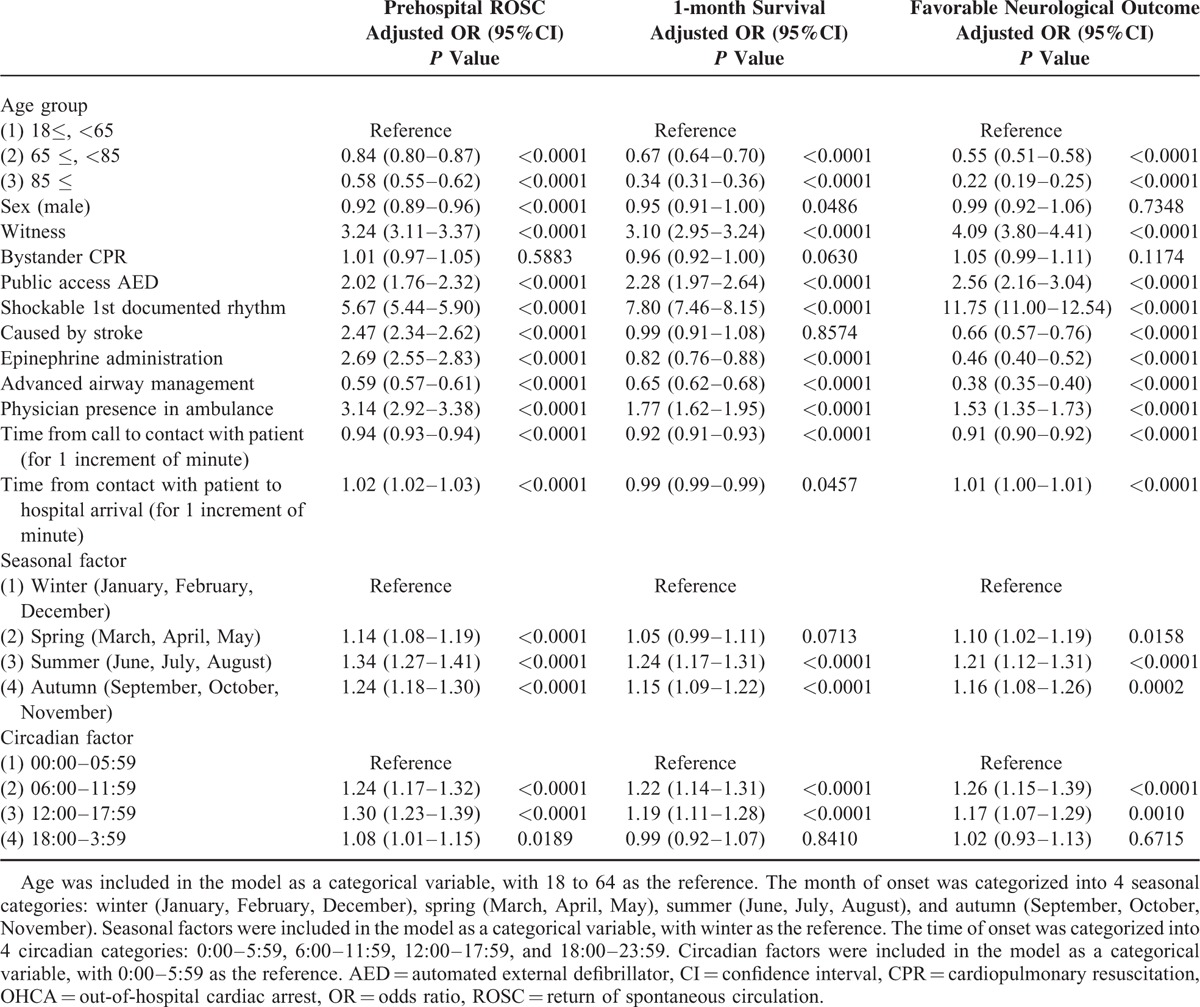
Factors Contributing to Favorable Outcomes in OHCA Patients Including Stroke Cases and Overall Presumed Cardiac Etiology

**TABLE 5:**
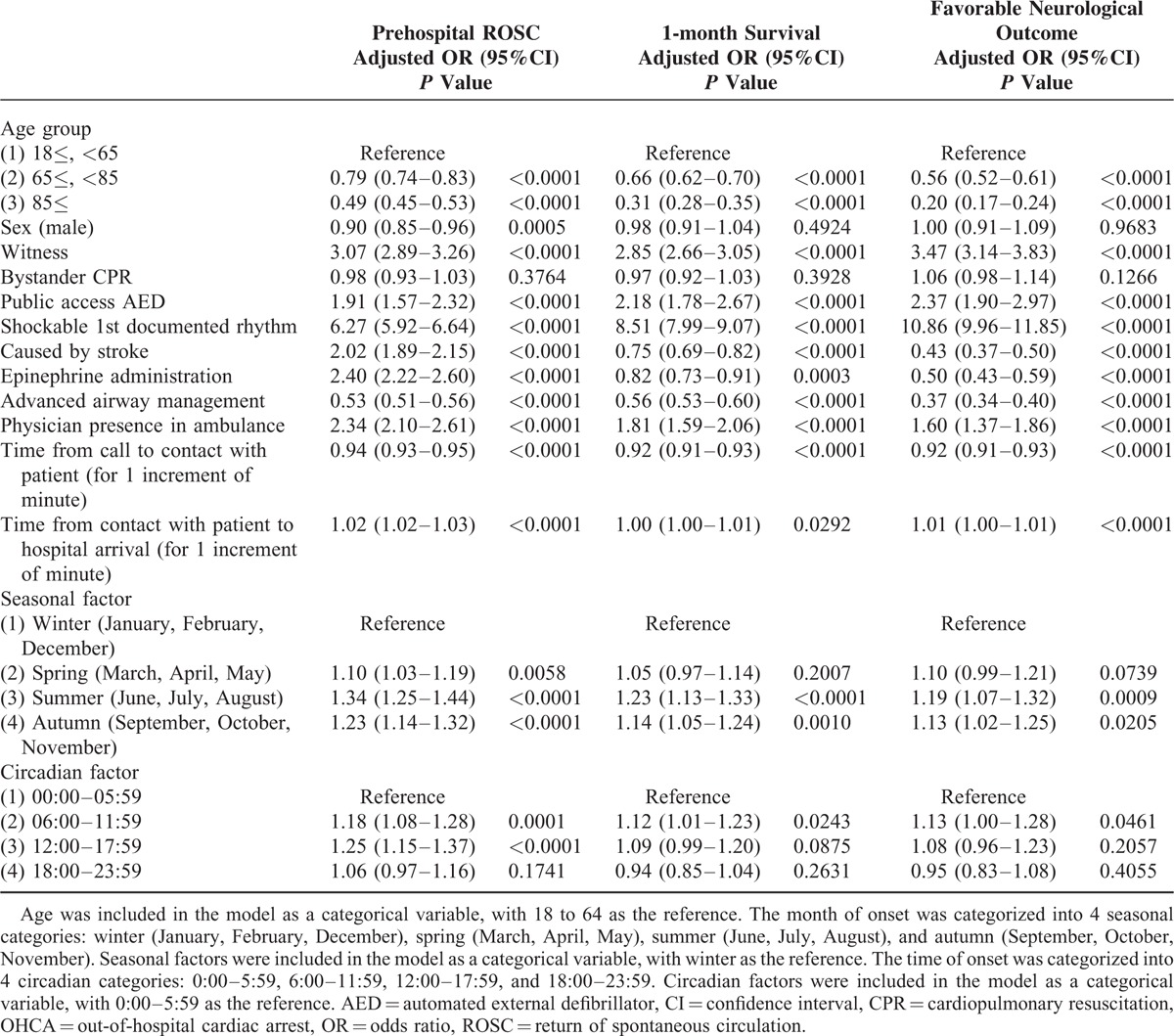
Factors Contributing to Favorable Outcomes in OHCA Patients Including Stroke Cases and Only Confirmed Cardiac Etiology

## DISCUSSION

In this nationwide, population-based study of OHCA patients with stroke etiology, we found that OHCA caused by stroke had a greater chance of prehospital ROSC but a lesser chance of 1-month survival or favorable neurological outcomes than OHCA caused by cardiac etiology. Being male and having a shockable initial rhythm were significantly associated with favorable neurological outcomes. In addition, prehospital advanced life support techniques (e.g., epinephrine administration or advanced airway management) were not associated with favorable neurological outcomes.

To the best of our knowledge, this study is the first to report the details of OHCA caused by stroke using a large sample size. Although several studies have reported on OHCA with stroke etiology, the largest cohort was only 86 patients.^10^ Therefore, only limited data have been available thus far.

The previous largest study concluded that resuscitative efforts for OHCA with neurological causes might be futile and that supportive treatments might be reoriented toward organ donation because the intensive care unit mortality rate was 100%. However, assuming that medical futility has a <1% chance of success in accordance with a quantitative approach,^20–22^ resuscitative efforts for OHCA with etiology of stroke may not be futile because the 1-month survival rate in our study was 3.6%, even after including unwitnessed, no bystander CPR, and nonshockable rhythm cases, as well as cases of elderly adults. In addition, the neurologically favorable survival rate was >1%. However, it cannot be ignored that neurological outcomes were much poorer in the stroke cases than in the cardiac etiology cases (particularly the confirmed cases). Thus, the cases in which favorable outcomes can be expected must be identified appropriately.

We investigated the factors that could contribute to favorable outcomes, and we made several useful observations. Younger age was associated with favorable outcomes in stroke-related OHCA, although age at onset was significantly lower in the stroke cases than in the cardiac etiology cases (Tables 1 and 3). In terms of sex differences, several studies have examined the impact of sex on stroke incidence and outcomes, and different studies have obtained differing results.^23–26^ However, in no other study except ours were the subjects limited to severe cases who had experienced sudden OHCA. In our study, the male–female ratio of cases of OHCA caused by stroke was 1:1, and we found no significant differences between sex and survival (adjusted OR 1.02, 95%CI 0.86–1.20). In terms of prehospital ROSC, the outcomes were poorer among men (adjusted OR 0.78, 95%CI 0.70–0.86), but their neurological outcomes were more favorable (adjusted OR 1.56, 95%CI 1.16–2.13). These differences in outcomes might reflect difference in physiological functions (such as endocrine and metabolic functions) between men and women. In addition, the sex differences between the incidence rates for each type of stroke (hemorrhagic or ischemic) might influence the outcomes. However, detailed data on these points were not available in this study. Further research is required to assess the association between sex difference and outcomes after stroke-related OHCA in more detail.

In terms of seasonal and circadian factors, our examination of OHCA cases (i.e., severe cases of stroke and cardiac disease) showed that onset was more frequent during the winter (January and December), early morning (6:00–9:59), and late afternoon (16:00–19:59), which is generally consistent with data previously reported in non-OHCA cases.^27–33^ In cardiac etiology cases (particularly confirmed cardiac etiology cases), patients had more favorable outcomes when the ambulance call occurred in the summer or autumn (June–October) or during the day (10:00–15:59), apparently in inverse proportion to the number of cases. For stroke cases, fixed tendencies were not observed between outcomes and seasonal or circadian factors.

In cases of OHCA caused by stroke, there was also an extremely strong relationship between shockable initial rhythm and favorable outcomes (favorable neurological outcome: adjusted OR 9.44, 95%CI 7.02–12.68; 1-month survival: adjusted OR 5.20, 95%CI 4.26–6.33; prehospital ROSC: adjusted OR 2.53, 95%CI 2.14–2.99). It is well known that stroke can cause fatal arrhythmia.^34–36^ Our results suggest that there are major differences between cases of OHCA caused by stroke depending on whether cardiac arrest is due to arrhythmia (including the trigeminocardiac reflex [TCR]^37,38^) or other causes (e.g., respiratory failure, heart failure, cerebral edema, etc.) and that there is a better chance of saving lives when arrhythmia is the cause.

Our finding that prehospital advanced life support did not contribute to favorable neurological outcomes is consistent with the results of past studies of OHCA of other etiologies.^39–41^ Prehospital advanced airway management was not beneficial for any outcome, but prehospital epinephrine administration and physician presence in the ambulance were associated with higher prehospital ROSC rates. However, these factors may merely constituted undue salvaging of patients who would have died otherwise.

The data that we examined did not include information on in-hospital or postresuscitation care, it only included prehospital care. Therefore, just as treatments such as percutaneous coronary intervention and targeted temperature management can definitively influence outcomes in cases of OHCA caused by cardiac etiology, to investigate whether superior treatments exist that can contribute to improving outcomes in cases of OHCA caused by stroke, data including information on in-hospital or postresuscitation care must be examined in detail.

Our study has some limitations. First, we analyzed all strokes together. The fundamental treatments differ for subarachnoid hemorrhage, intracerebral hemorrhage, and cerebral infarction; therefore, it would be best to examine them separately. However, we were unable to obtain data on the patient ratios for these diseases. In addition, head trauma should have been categorized under external causes, but some cases might have been categorized as strokes.

Second, the true cause of death was unclear. For OHCA caused by stroke, we can only know whether acute stroke was present when cardiac arrest occurred. The real cause that led to ultimate death might not be brain damage itself but rather respiratory failure or arrhythmia (including the TCR) due to stroke.

Third, Japan has a low autopsy rate, therefore, the true cause of death is sometimes unidentified. In addition, the recorded diagnoses may not be definitive. If there were no evidence supporting a particular diagnosis, cases were categorized as presumed cardiac etiology. Therefore, some undiagnosed stroke cases might be categorized under presumed cardiac etiology. In addition, some cases that should have been categorized as presumed cardiac etiology might be categorized under noncardiac etiology.

Fourth, the All-Japan Utstein Registry of the FDMA does not collect information on every aspect of resuscitation care. It includes information only on prehospital care, not in-hospital or postresuscitation care. It also does not include the patients past histories, including underlying diseases and medications. The information regarding prehospital care is only quantitative data, not qualitative data. In addition, although in-hospital or postresuscitation care may seriously influence outcomes (e.g., brain surgery, cardiac catheterization, thrombolytics, anticoagulants, antiplatelets, extracorporeal life support, targeted temperature management, or do-not-resuscitate orders), these data are not available. The possibility of residual confounding remains.

Fifth, the findings of this study might only indicate association and not causality. Although this study was conducted under the assumption that the same resuscitation efforts were attempted in all registered patients, the same resuscitation efforts might not be made for all OHCA patients. The awareness of OHCA etiology might affect the quality of in-hospital or postresuscitation care.

Finally, as with all epidemiological studies, the data integrity, validity, and ascertainment bias are potential limitations. The use of a uniform data record based on the Utstein-style guidelines for reporting cardiac arrest, the large sample size, and the population-based design were intended to minimize these potential sources of bias.

## CONCLUSIONS

In conclusion, in a nationwide, population-based study, we found that OHCA with an etiology of stroke had lower 1-month survival rates and poorer neurological outcomes than OHCA with a cardiac etiology. However, the results were not so dire to be considered medically futile. Men and patients with a shockable initial rhythm may have a greater chance of survival with a favorable neurological outcome. However, it appears that prehospital advanced life support does not improve the neurological outcomes. More research, including in-hospital data gathering, must be performed to find superior treatments for OHCA caused by stroke.

## Supplementary Material

Supplemental Digital Content

## References

[1] World Health Organization. The top 10 causes of death. Geneva: WHO Available at http://www.who.int/mediacentre/factsheets/fs310/en/ Accessed March 25, 2016.

[2] Sörös P Hachinski V Cardiovascular and neurological causes of sudden death after ischaemic stroke. *Lancet Neurol* 2012; 11:179–188. 22265213 10.1016/S1474-4422(11)70291-5

[3] Solomon SD Zelenkofske S McMurray JJ Valsartan in Acute Myocardial Infarction Trial (VALIANT) Investigators. Sudden death in patients with myocardial infarction and left ventricular dysfunction, heart failure, or both. *N Engl J Med* 2005; 352:2581–2588. 15972864 10.1056/NEJMoa043938

[4] Adabag AS Therneau TM Gersh BJ Sudden death after myocardial infarction. *JAMA* 2008; 300:2022–2029. 18984889 10.1001/jama.2008.553 PMC2731625

[5] Spaulding CM Joly LM Rosenberg A Immediate coronary angiography in survivors of out-of-hospital cardiac arrest. *N Engl J Med* 1997; 336:1629–1633. 9171064 10.1056/NEJM199706053362302

[6] Buxton AE Lee KL Fisher JD A randomized study of the prevention of sudden death in patients with coronary artery disease. Multicenter Unsustained Tachycardia Trial Investigators. *N Engl J Med* 1999; 341:1882–1890. 10601507 10.1056/NEJM199912163412503

[7] Hypothermia after Cardiac Arrest Study Group. Mild therapeutic hypothermia to improve the neurologic outcome after cardiac, arrest. *N Engl J Med* 2002; 346:549–556. 11856793 10.1056/NEJMoa012689

[8] Nielsen N Wetterslev J Cronberg T TTM Trial Investigators. Targeted temperature management at 33 °C versus 36 °C after cardiac arrest. *N Engl J Med* 2013; 369:2197–2206. 24237006 10.1056/NEJMoa1310519

[9] Chen YS Lin JW Yu HY Cardiopulmonary resuscitation with assisted extracorporeal life-support versus conventional cardiopulmonary resuscitation in adults with in-hospital cardiac arrest: an observational study and propensity analysis. *Lancet* 2008; 372:554–561. 18603291 10.1016/S0140-6736(08)60958-7

[10] Arnaout M Mongardon N Deye N Out-of-hospital cardiac arrest from brain cause: epidemiology, clinical features, and outcome in a multicenter cohort^∗^. *Crit Care Med* 2015; 43:453–460. 25599468 10.1097/CCM.0000000000000722

[11] Kitamura T Kiyohara K Sakai T Epidemiology and outcome of adult out-of-hospital cardiac arrest of non-cardiac origin in Osaka: a population-based study. *BMJ Open* 2014; 4:e006462. 10.1136/bmjopen-2014-006462 PMC4275684 25534213

[12] Ro YS Shin SD Song KJ A comparison of outcomes of out-of-hospital cardiac arrest with non-cardiac etiology between emergency departments with low- and high-resuscitation case volume. *Resuscitation* 2012; 83:855–861. 22366719 10.1016/j.resuscitation.2012.02.002

[13] Dumas F Farhenbruch C Hambly C Predicting non-cardiac aetiology: a strategy to allocate rescue breathing during bystander CPR. *Resuscitation* 2012; 83:134–137. 21983124 10.1016/j.resuscitation.2011.09.022

[14] Fukuda T Fukuda-Ohashi N Doi K Effective pre-hospital care for out-of-hospital cardiac arrest caused by respiratory disease. *Heart Lung Circ* 2015; 24:241–249. 25445432 10.1016/j.hlc.2014.09.004

[15] Cummins RO Chamberlain D Hazinski MF Recommended guidelines for reviewing, reporting, and conducting research on in-hospital resuscitation: the in-hospital ‘Utstein style’. American Heart Association. *Circulation* 1997; 95:2213–2239. 9133537 10.1161/01.cir.95.8.2213

[16] Jacobs I Nadkarni V Bahr J International Liaison Committee on Resuscitation; American Heart Association; European Resuscitation Council; Australian Resuscitation Council; New Zealand Resuscitation Council; Heart and Stroke Foundation of Canada; InterAmerican Heart Foundation; Resuscitation Councils of Southern Africa; ILCOR Task Force on Cardiac Arrest and Cardiopulmonary Resuscitation Outcomes. Cardiac arrest and cardiopulmonary resuscitation outcome reports: update and simplification of the Utstein templates for resuscitation registries: a statement for healthcare professionals from a task force of the International Liaison Committee on Resuscitation (American Heart Association, European Resuscitation Council, Australian Resuscitation Council, New Zealand Resuscitation Council, Heart and Stroke Foundation of Canada, InterAmerican Heart Foundation, Resuscitation Councils of Southern Africa). *Circulation* 2004; 110:3385–3397. 15557386 10.1161/01.CIR.0000147236.85306.15

[17] Fukuda T Matsubara T Doi K Predictors of favorable and poor prognosis in unwitnessed out-of-hospital cardiac arrest with a non-shockable initial rhythm. *Int J Cardiol* 2014; 176:910–915. 25168100 10.1016/j.ijcard.2014.08.057

[18] Fukuda T Ohashi-Fukuda N Matsubara T Trends in outcomes for out-of-hospital cardiac arrest by age in Japan: an observational study. *Medicine (Baltimore)* 2015; 94:e2049. 26656330 10.1097/MD.0000000000002049 PMC5008475

[19] Jennett B Bond M Assessment of outcome after severe brain damage. *Lancet* 1975; 1:480–484. 46957 10.1016/s0140-6736(75)92830-5

[20] Schneiderman LJ Jecker NS Jonsen AR Medical futility: its meaning and ethical implications. *Ann Intern Med* 1990; 112:949–954. 2187394 10.7326/0003-4819-112-12-949

[21] Helft PR Siegler M Lantos J The rise and fall of the futility movement. *N Engl J Med* 2000; 343:293–296. 10911014 10.1056/NEJM200007273430411

[22] Cantor MD Braddock CH 3rd Derse AR Veterans Health Administration National Ethics Committee. Do-not-resuscitate orders and medical futility. *Arch Intern Med* 2003; 163:2689–2694. 14662622 10.1001/archinte.163.22.2689

[23] Reeves MJ Bushnell CD Howard G Sex differences in stroke: epidemiology, clinical presentation, medical care, and outcomes. *Lancet Neurol* 2008; 7:915–926. 18722812 10.1016/S1474-4422(08)70193-5 PMC2665267

[24] Gall SL Donnan G Dewey HM Sex differences in presentation, severity, and management of stroke in a population-based study. *Neurology* 2010; 74:975–981. 20181922 10.1212/WNL.0b013e3181d5a48f

[25] Appelros P Stegmayr B Terént A Sex differences in stroke epidemiology: a systematic review. *Stroke* 2009; 40:1082–1090. 19211488 10.1161/STROKEAHA.108.540781

[26] Gargano JW Wehner S Reeves M Sex differences in acute stroke care in a statewide stroke registry. *Stroke* 2008; 39:24–249. 18048851 10.1161/STROKEAHA.107.493262

[27] Nyquist PA Brown RD Jr Wiebers DO Circadian and seasonal occurrence of subarachnoid and intracerebral hemorrhage. *Neurology* 2001; 56:190–193. 11160954 10.1212/wnl.56.2.190

[28] Rothwell PM Wroe SJ Slattery J Is stroke incidence related to season or temperature? The Oxfordshire Community Stroke Project. *Lancet* 1996; 347:934–936. 8598757 10.1016/s0140-6736(96)91415-4

[29] Wroe SJ Sandercock P Bamford J Diurnal variation in incidence of stroke: Oxfordshire community stroke project. *BMJ* 1992; 304:155–157. 1737160 10.1136/bmj.304.6820.155 PMC1881192

[30] Argentino C Toni D Rasura M Circadian variation in the frequency of ischemic stroke. *Stroke* 1990; 21:387–389. 2309262 10.1161/01.str.21.3.387

[31] Elliott WJ Circadian variation in the timing of stroke onset: a meta-analysis. *Stroke* 1998; 29:992–996. 9596248 10.1161/01.str.29.5.992

[32] Marchant B Ranjadayalan K Stevenson R Circadian and seasonal factors in the pathogenesis of acute myocardial infarction: the influence of environmental temperature. *Br Heart J* 1993; 69:385–387. 8518058 10.1136/hrt.69.5.385 PMC1025097

[33] Spielberg C Falkenhahn D Willich SN Circadian, day-of-week, and seasonal variability in myocardial infarction: comparison between working and retired patients. *Am Heart J* 1996; 132:579–585. 8800028 10.1016/s0002-8703(96)90241-0

[34] Koppikar S Baranchuk A Guzmán JC Stroke and ventricular arrhythmias. *Int J Cardiol* 2013; 168:653–659. 23602297 10.1016/j.ijcard.2013.03.058

[35] Katsanos AH Korantzopoulos P Tsivgoulis G Electrocardiographic abnormalities and cardiac arrhythmias in structural brain lesions. *Int J Cardiol* 2013; 167:328–334. 22809542 10.1016/j.ijcard.2012.06.107

[36] Oppenheimer SM Cechetto DF Hachinski VC Cerebrogenic cardiac arrhythmias. Cerebral electrocardiographic influences and their role in sudden death. *Arch Neurol* 1990; 47:513–519. 2185720 10.1001/archneur.1990.00530050029008

[37] Chowdhury T Mendelowith D Golanov E Trigemino-Cardiac Reflex Examination Group. Trigeminocardiac reflex: the current clinical and physiclogical knoeledge. *J Neurosurg Anesthesiol* 2015; 27:136–147. 25602626 10.1097/ANA.0000000000000065

[38] Spiriev T Kondoff S Schaller B Trigemino-Cardiac-Reflex-Examination-Group. Cardiovascular changes after subarachnoid hemorrhage initiated by the trigeminocardiac reflex – first description of a case series. *J Neurosurg Anesthesiol* 2011; 23:379–380. 21908992 10.1097/ANA.0b013e3182312486

[39] Sanghavi P Jena AB Newhouse JP Outcomes after out-of-hospital cardiac arrest treated by basic vs advanced life support. *JAMA Intern Med* 2015; 175:196–204. 25419698 10.1001/jamainternmed.2014.5420 PMC4314335

[40] Hasegawa K Hiraide A Chang Y Association of prehospital advanced airway management with neurologic outcome and survival in patients with out-of-hospital cardiac arrest. *JAMA* 2013; 309:257–266. 23321764 10.1001/jama.2012.187612

[41] Hagihara A Hasegawa M Abe T Prehospital epinephrine use and survival among patients with out-of-hospital cardiac arrest. *JAMA* 2012; 307:1161–1168. 22436956 10.1001/jama.2012.294

