# Supplementary material for: Epidemiology, Risk Factors, and Outcomes of Out-of-Hospital Cardiac Arrest Caused by Stroke: A Population-Based Study
Source: Medicine (Baltimore). 2016 Apr 8;95(14):e3107. doi: 10.1097/MD.0000000000003107 (PMC4998750; doi:10.1097/MD.0000000000003107)
Supplement: Supplemental Digital Content [file medi-95-e3107-s001.doc]

**Appendix 1. Occurrence of OHCA according to each etiology by month or time of onset (from Jan. 2006 to Dec. 2009)**

The data are expressed as the number (%), unless otherwise indicated.

**Appendix 2. One-month survival rates and favorable neurological outcomes for OHCA, according to each etiology by month or time of onset** **(from Jan. 2006 to Dec. 2009)**

The data are expressed as the number (%), unless otherwise indicated.

**Appendix 3. Occurrence of witnessed OHCA according to each etiology by month or time of onset (from Jan. 2006 to Dec. 2009)**

The data are expressed as the number (%), unless otherwise indicated.

**Appendix 4. One-month survival rates and favorable neurological outcomes for witnessed OHCA, according to each etiology by month or time of onset (from Jan. 2006 to Dec. 2009)**

The data are expressed as the number (%), unless otherwise indicated.
